# Supplementary material for: EcR recruits dMi-2 and increases efficiency of dMi-2-mediated remodelling to constrain transcription of hormone-regulated genes
Source: Nat Commun. 2017 Apr 5;8:14806. doi: 10.1038/ncomms14806 (PMC5382322; doi:10.1038/ncomms14806)
Supplement: Supplementary Information — Supplementary Figures and Supplementary Tables [file ncomms14806-s1.pdf]

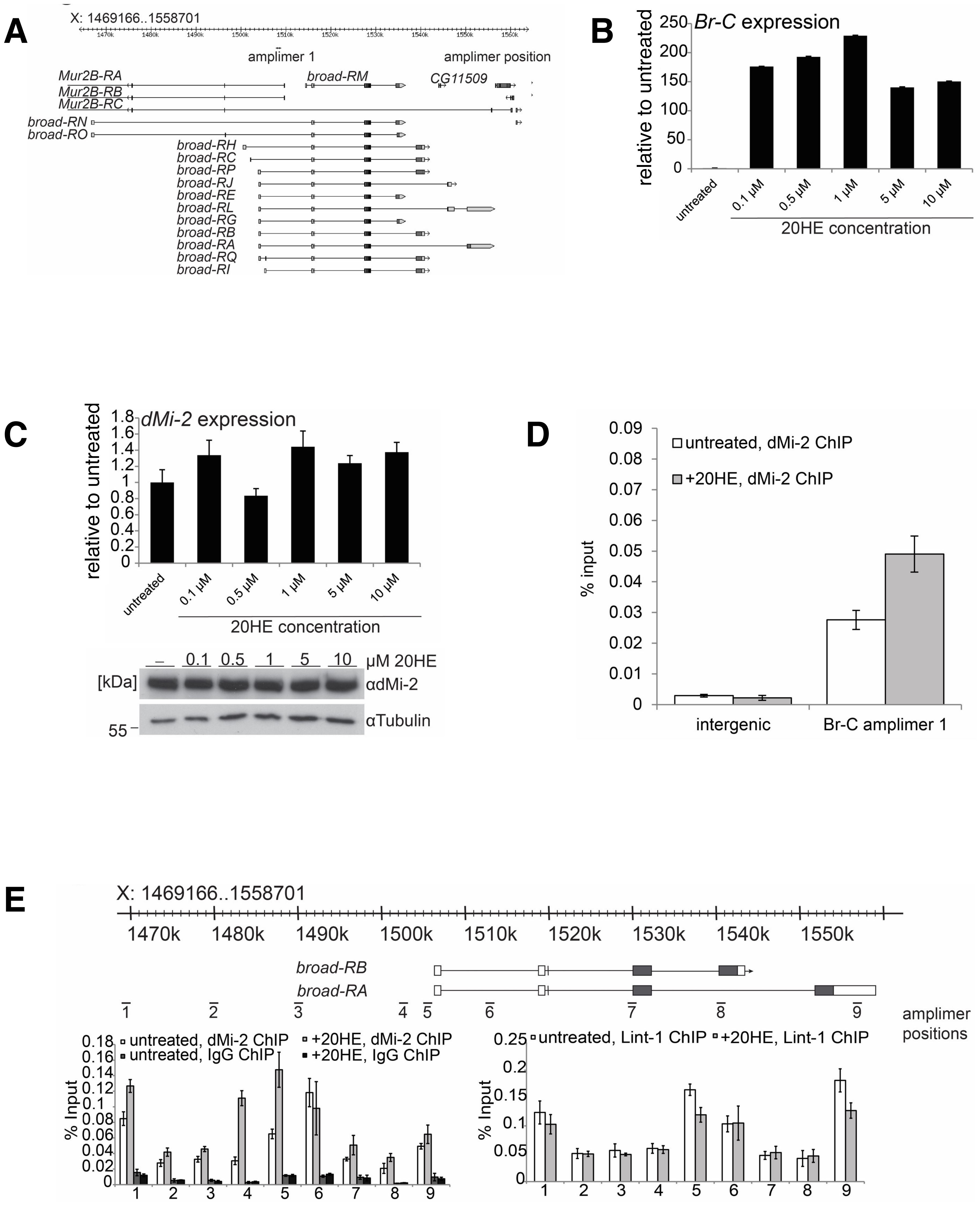

Supplementary Figure 1

F

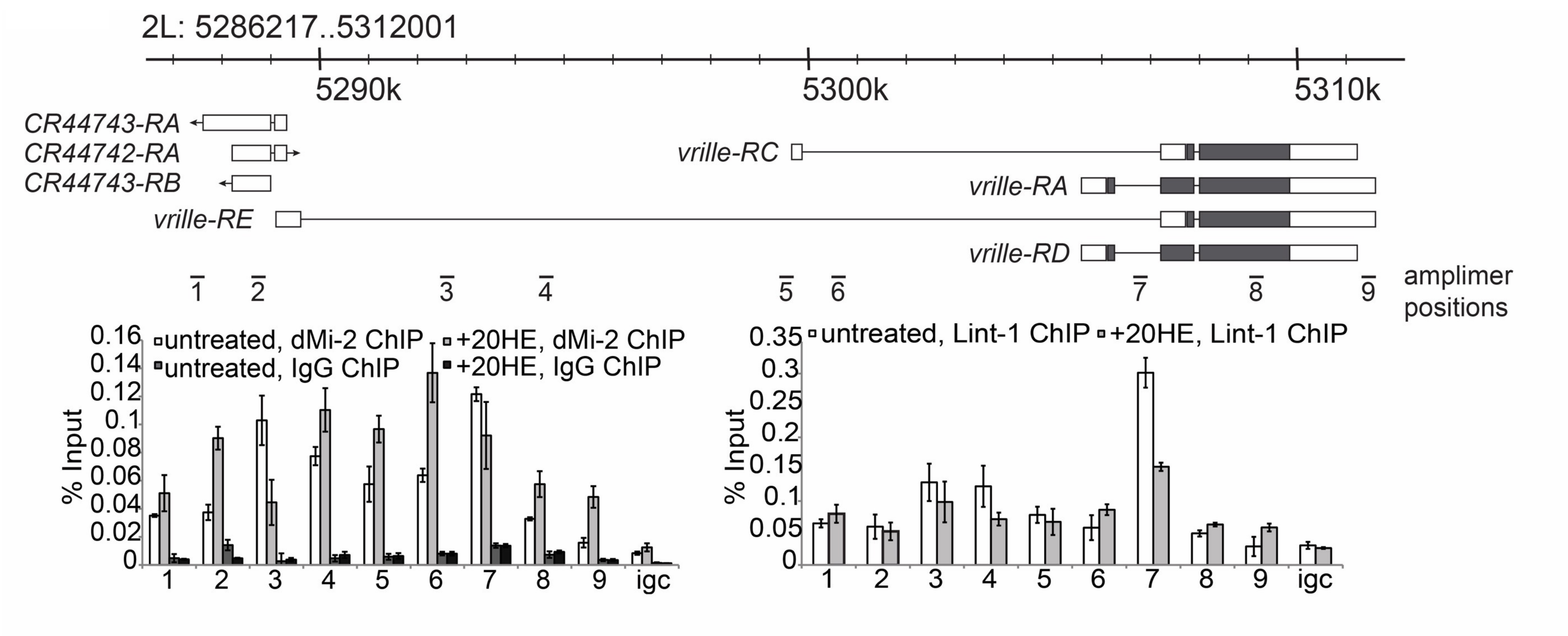

G

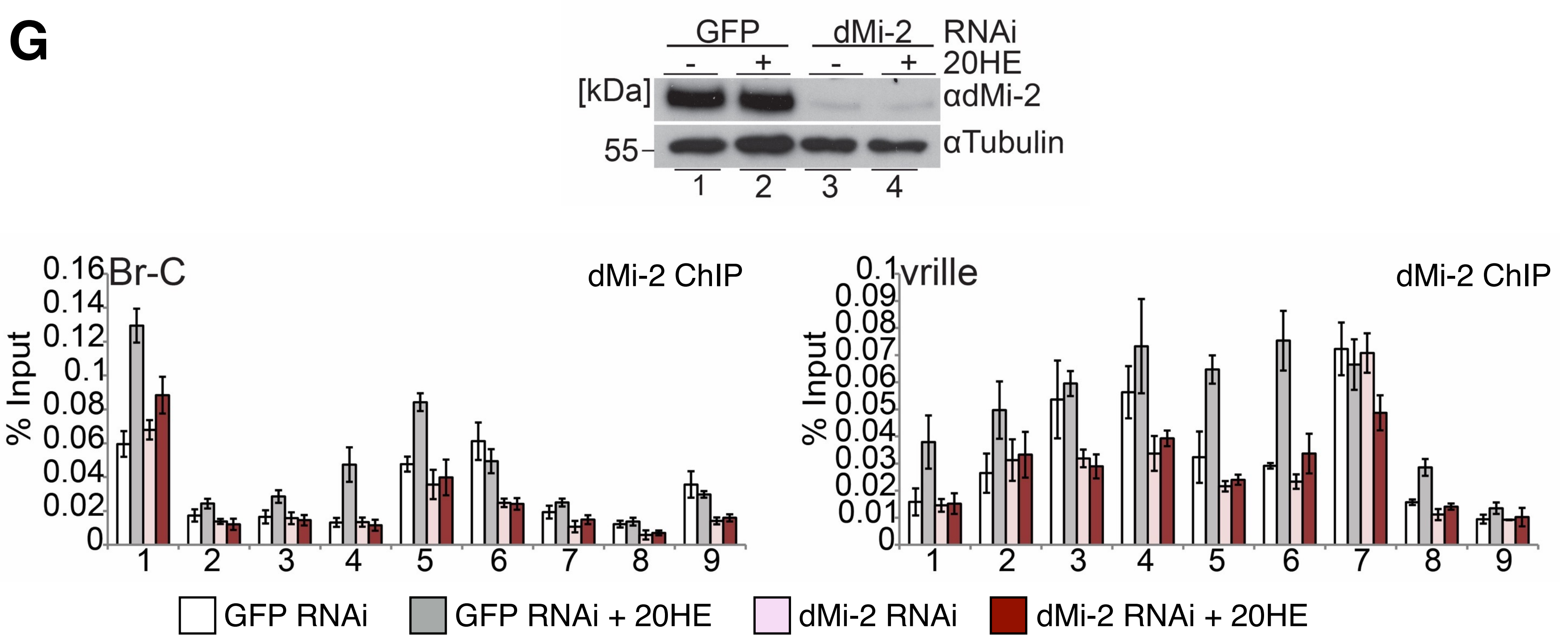

Supplementary Figure 1. dMi-2 binds ecdysone-activated genes.
(A) Schematic representation of the *Br-C* gene locus. Amplimer position within the first intron of the major *Br-C* transcripts used for RT-qPCR and ChIP is indicated.
(B) Analysis of *Br-C* RNA expression in S2 cells treated with 20-hydroxy ecdysone (20HE) for 6 hours determined by RT-qPCR. The ratios of *Br-C* to *rp49* RNA levels in untreated and 20HE-treated cells were calculated. The ratio determined in untreated cells was set to 1 and other ratios were expressed relative to this. Concentrations of 20HE used are indicated below the figure. Error bars denote standard deviation of technical triplicates.
(C) Left panel: Analysis of *dMi-2* RNA expression in S2 cells treated with 20-hydroxy ecdysone (20HE) for 6 hours determined by RT-qPCR. The ratios of *dMi-2* to *rp49* RNA levels in untreated and 20HE-treated cells were calculated. The ratio determined in untreated cells was set to 1 and other ratios were expressed relative to this. Concentrations of 20HE used are indicated below the figure. Error bars denote standard deviation of technical triplicates. Right panel: Protein extracts from S2 cells treated with 20HE as described above were analysed by Western blot using the antibodies indicated on the right.
(D) Chromatin was prepared from untreated and 20HE treated S2 cells. ChIP was performed with dMi-2 antibody. Immunoprecipitated DNA was quantified by qPCR by amplifying a region located within the first intron of the major *Br-C* transcripts (*Br-C* amplimers 1; see Figure S1A) and an intergenic region on chromosome arm 2R (see Experimental Procedures). Error bars denote standard deviation of technical triplicates.
(E) dMi-2 but not Lint-1 is recruited to the *Br-C* locus upon ecdysone treatment. Top: schematic representation of *Br*-C locus indicating positions of amplimers used for ChIP-qPCR. Chromatin was prepared from untreated and 20HE treated S2 cells. ChIP was performed with dMi-2 (middle panel) or dLint (bottom panel) antibodies. Error bars denote standard deviation of technical triplicates. Experiments were performed as biological triplicates. One representative example is shown.
(F) dMi-2 but not Lint-1 is recruited to the *vrille* locus upon ecdysone treatment. Top: schematic representation of *vrille* locus indication positions of amplimers used for ChIP-qPCR. Chromatin was prepared from untreated and 20HE treated S2 cells. ChIP was performed with dMi-2 (middle panel) or dLint (bottom panel) antibodies. Error bars denote standard deviation of technical triplicates. Experiments were performed as biological triplicates. One representative example is shown.
(G) dMi-2 RNAi depletion abrogates recruitment to *Br-C* and *vrille*. Top: Western blot analysis of protein extracts of S2 cells treated with dsRNA directed against GFP (control; GFP RNAi) or dMi-2 (dMi-2 RNAi) message. Cells were either left untreated (- 20HE) or treated with ecdysone (+ 20HE). Antibodies used are shown on the right, molecular masses on the left. Tubulin was analysed as a loading control. Bottom panels: ChIP-qPCR following dMi-2 depletion. Chromatin was prepared from S2 cells treated with dsRNA and/or ecdysone as indicated. ChIP was performed with dMi-2 antibody. Regions spanning the *Br*-C (left panel) and vrille (right panel) were amplified by qPCR. See Figure S2E and S2F for amplimer positions. Error bars denote standard deviation of technical triplicates. Experiments were performed as biological triplicates. One representative example is shown.

Supplementary Figure 1

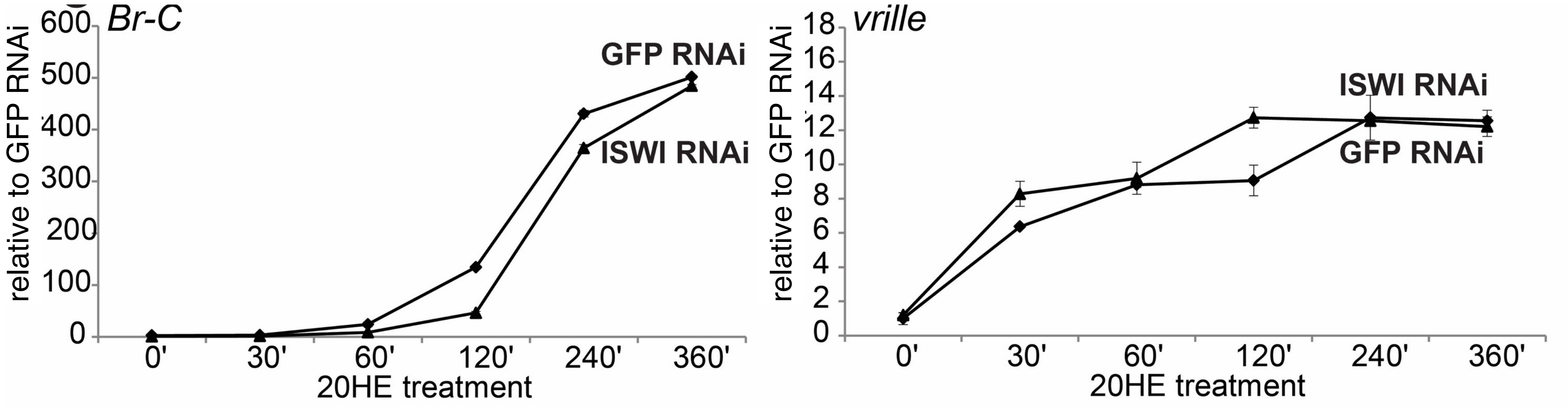

Supprementary Figure 2 dMi-2 contributes to a closed chromatin structure and limits the expression of ecdysone-activated genes. Time course of *Br*-C (left panel) and *vrille* (right panel) RNA expression over 6 hours of ecdysone treatment. Prior to ecdysone addition cells were depleted of GFP (control) or ISWI by RNAi as indicated. *Br*-C and vrille RNA levels were determined by RT-qPCR at the time points indicated.The ratios of *Br*-C and *vrille* RNA levels to *rp49* RNA levels were calculated and plotted. The ratio determined in untreated cells (time = 0 minutes) was set to 1 and other ratios were expressed relative to this.

Supplementary Figure 2

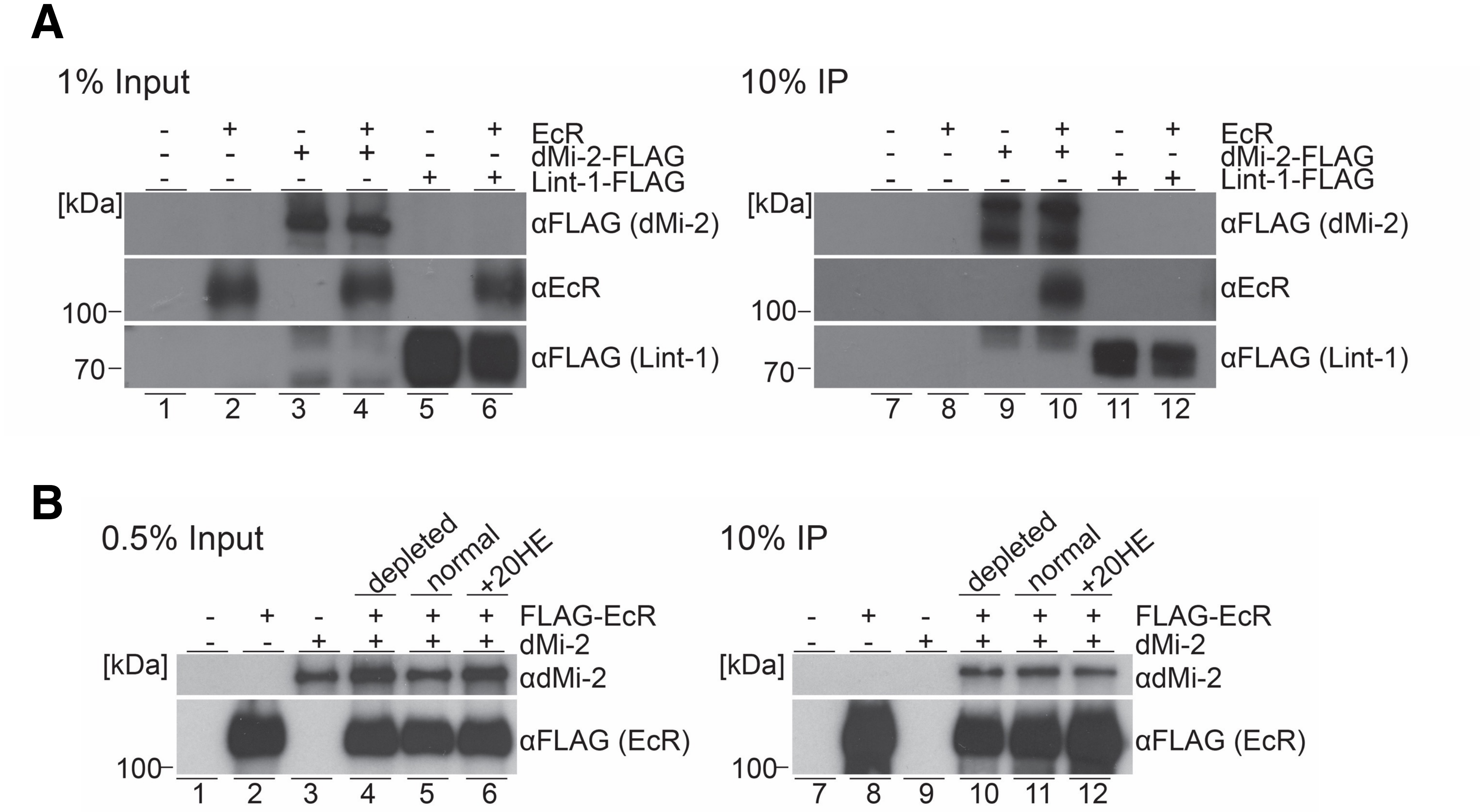

Supplementary Figure 3: dMi-2 forms a complex with EcR and competes with USP for binding to EcR.

(A) Sf9 cells were left untreated or were infected with recombinant baculoviruses directing expression of EcR, dMi-2-FLAG or dLint-FLAG as indicated on top. Extracts were immunoprecipitated with FLAG antibody. Protein extracts (left panel, 1% input) and immunoprecipitates (right panel, 10% IP) were probed by Western blot with FLAG and EcR antibodies as indicated on the right. Molecular masses are indicated on the left.

(B) Interaction of Mi-2 with EcR is independent of 20HE. Sf9 cells were infected with baculoviruses expressing FLAG-EcR and untagged Mi-2 as indicated on top in charcoal-treated (hormone depleted), normal or 20HE supplemented medium. Extracts were immunoprecipitated with FLAG antibody. Immunoprecipitates were subjected to Western blot using antibodies indicated on the right. Left panel shows 0.5% of the total input sample that was used for IP. Right panel shows 10% of the total immunoprecipitated sample. Molecular masses are depicted on the left.

Supplementary Figure 3

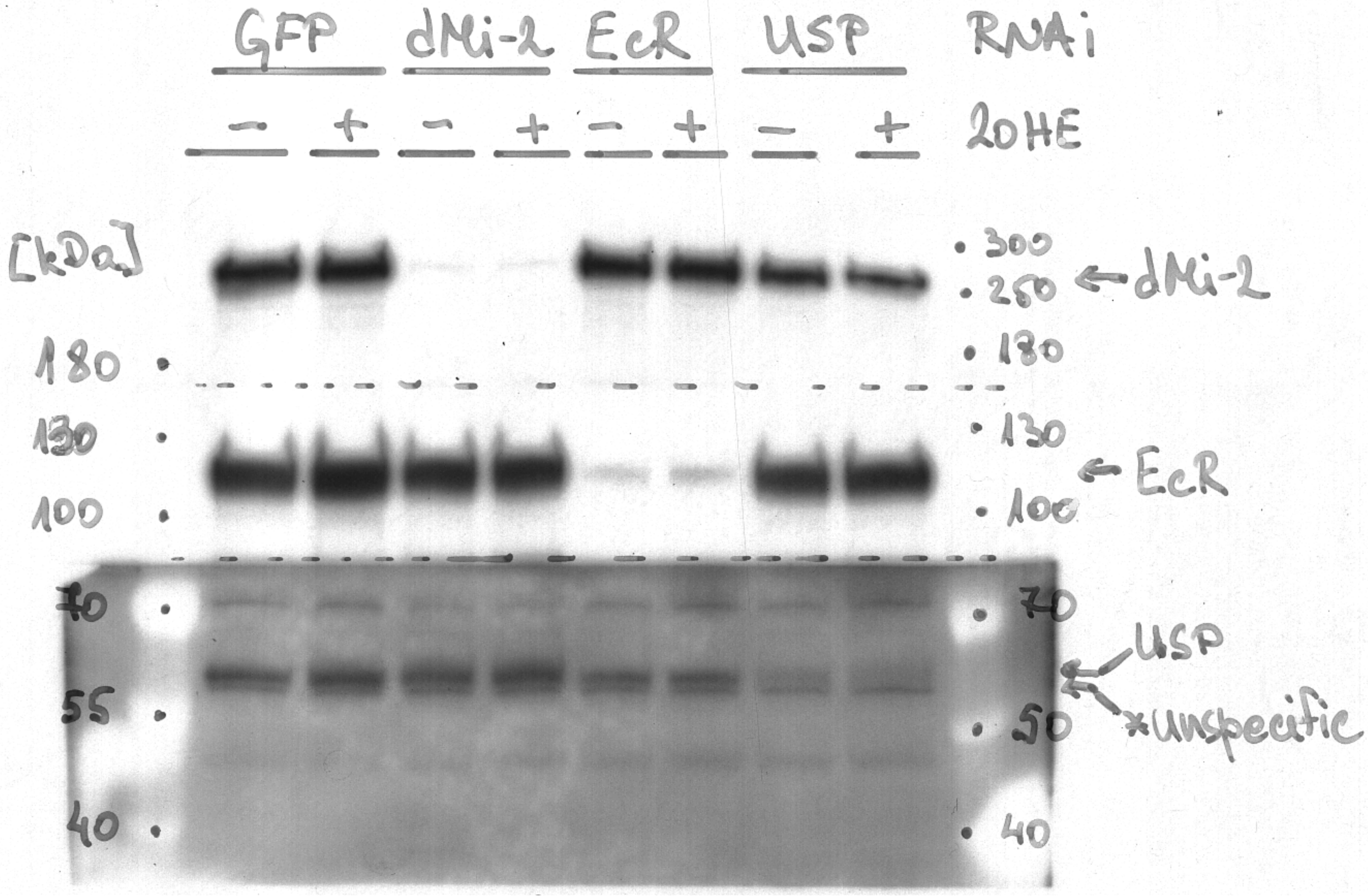

Figure 2A: Uncropped Western Blot
(membrane was cut as indicated by striped line)

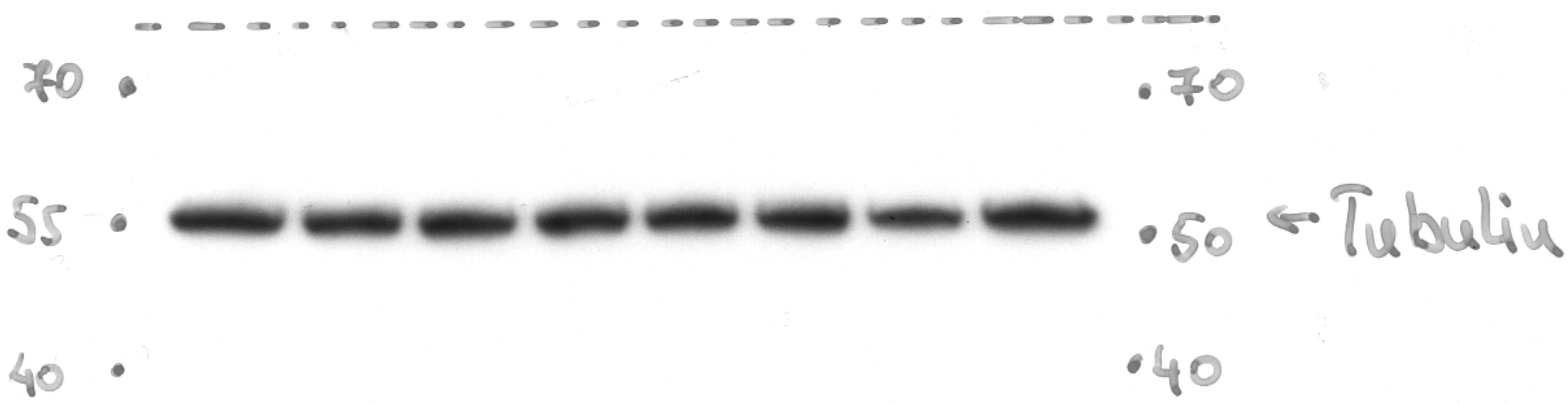

Figure 2A: Uncropped Western Blot
(lower part of membrane was stripped and
reprobed with anti-tubulin)

Supplementary Figure 4

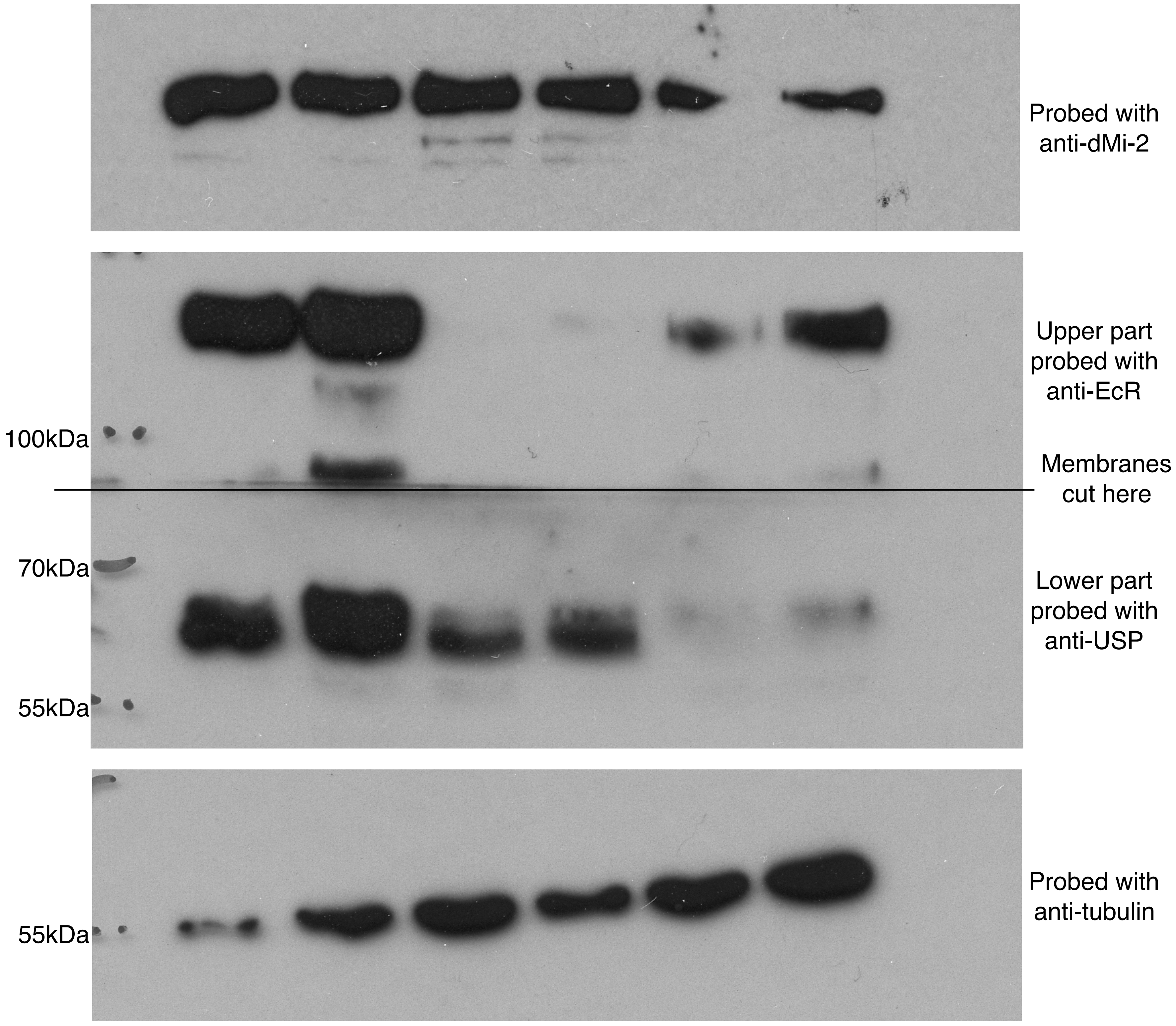

Figure 3B: Uncropped Western Blot

Supplementary Figure 4

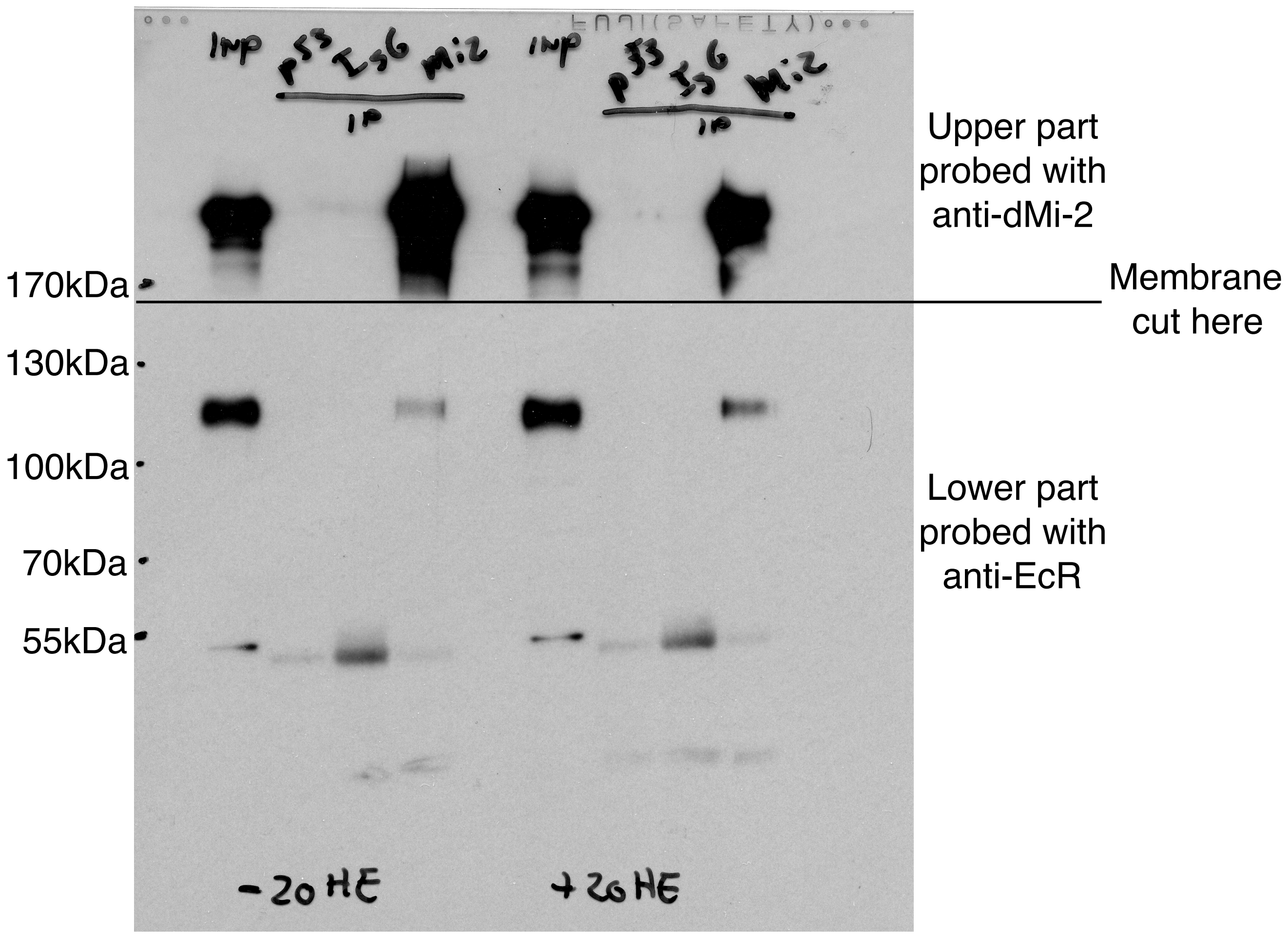

Figure 4A: Uncropped Western Blot

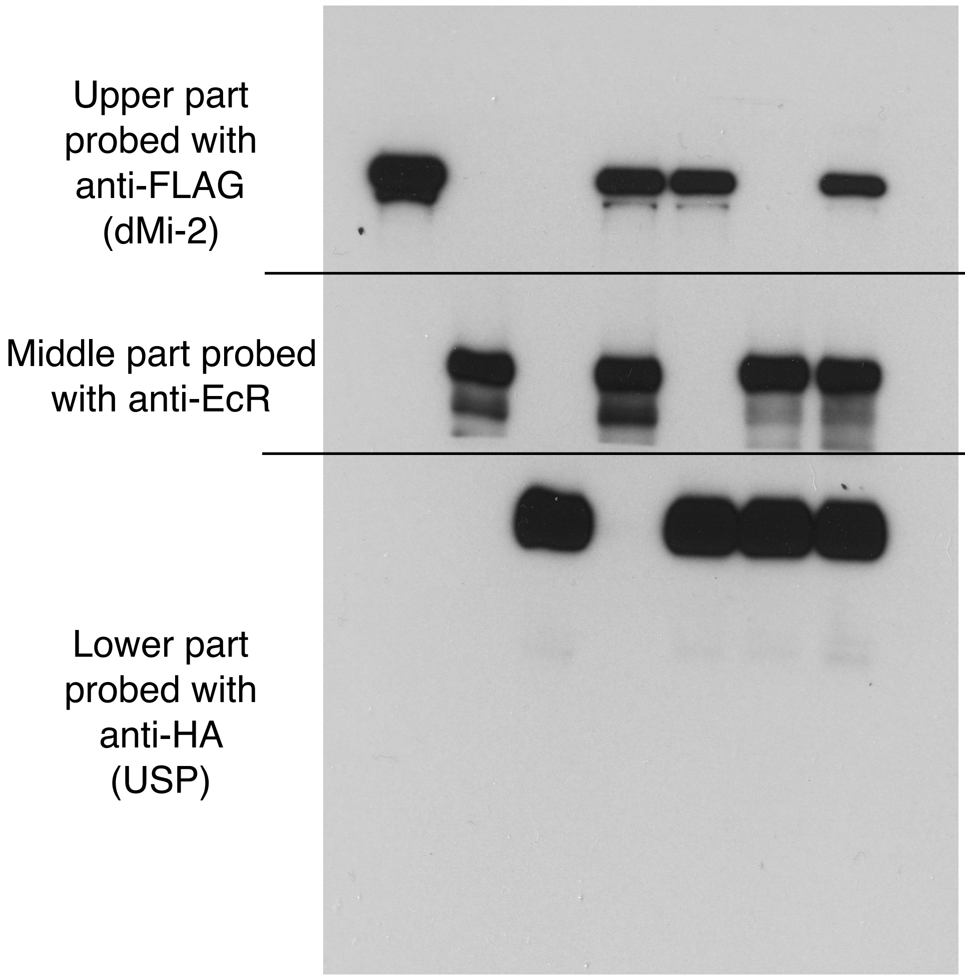

Figure 4C: Uncropped Western Blot, Input, upper left

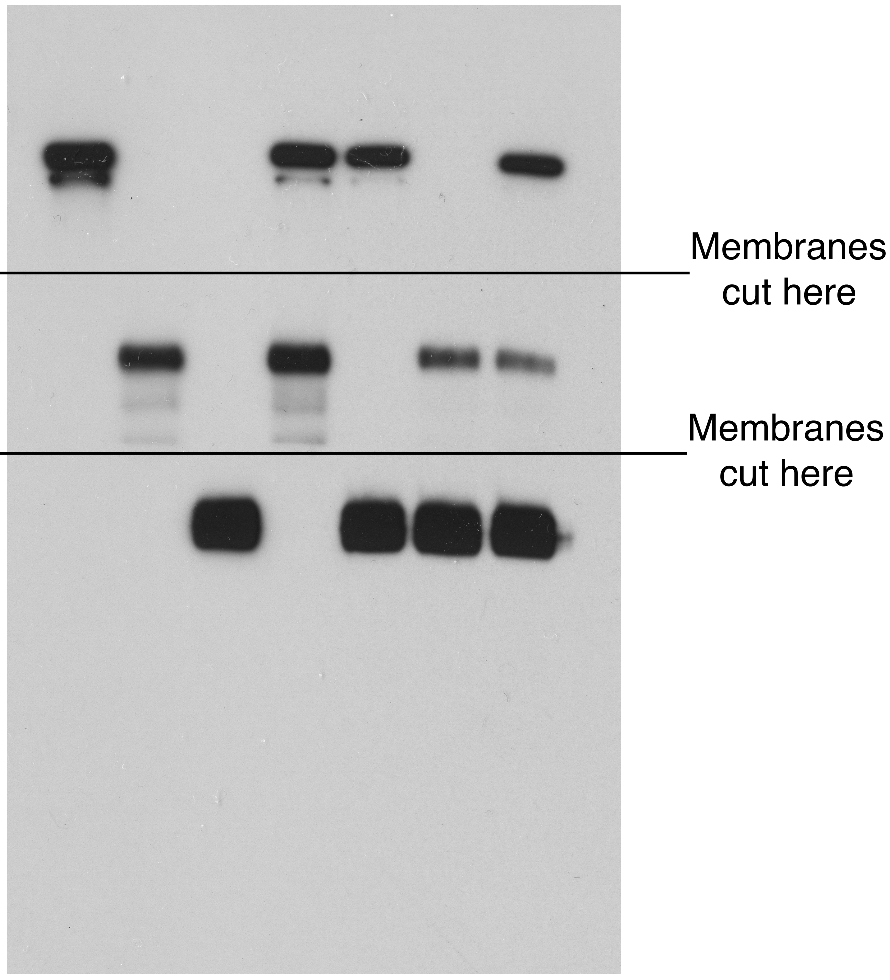

Figure 4C: Uncropped Western Blot, Input, upper right

Supplementary Figure 4

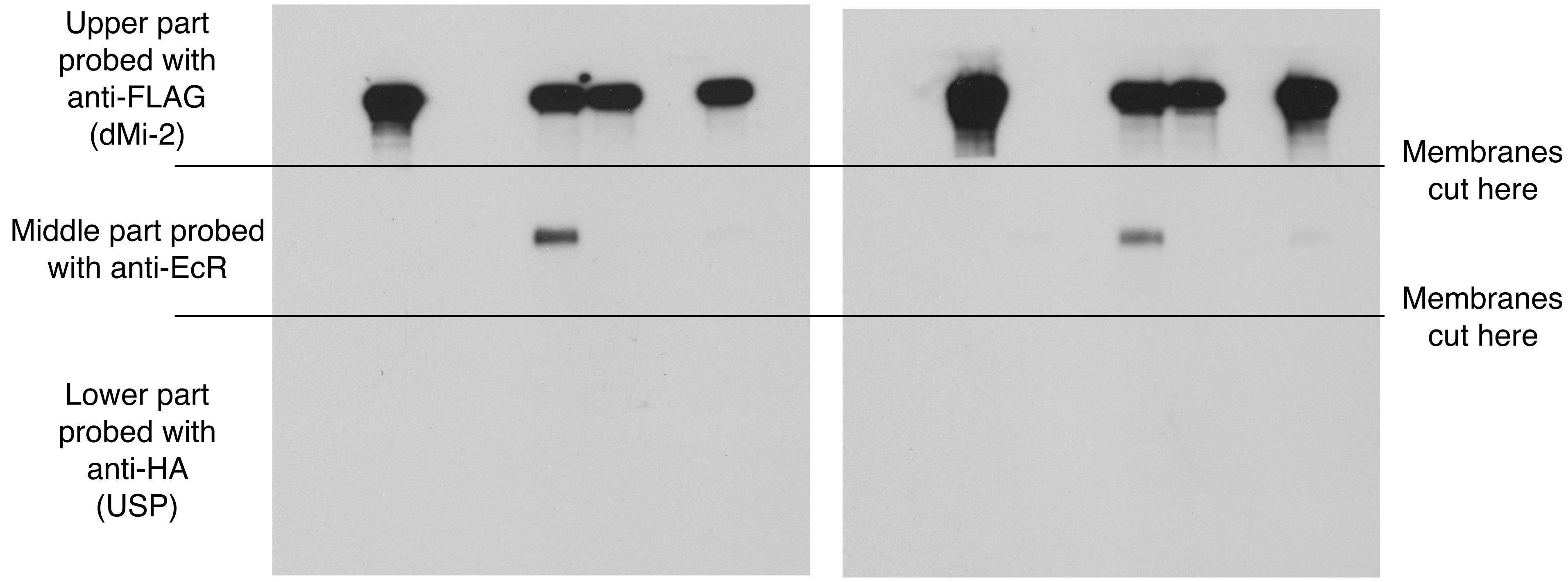

Figure 4C: Uncropped Western Blot, FLAG IP, middle left

Figure 4C: Uncropped Western Blot, FLAG IP, middle right

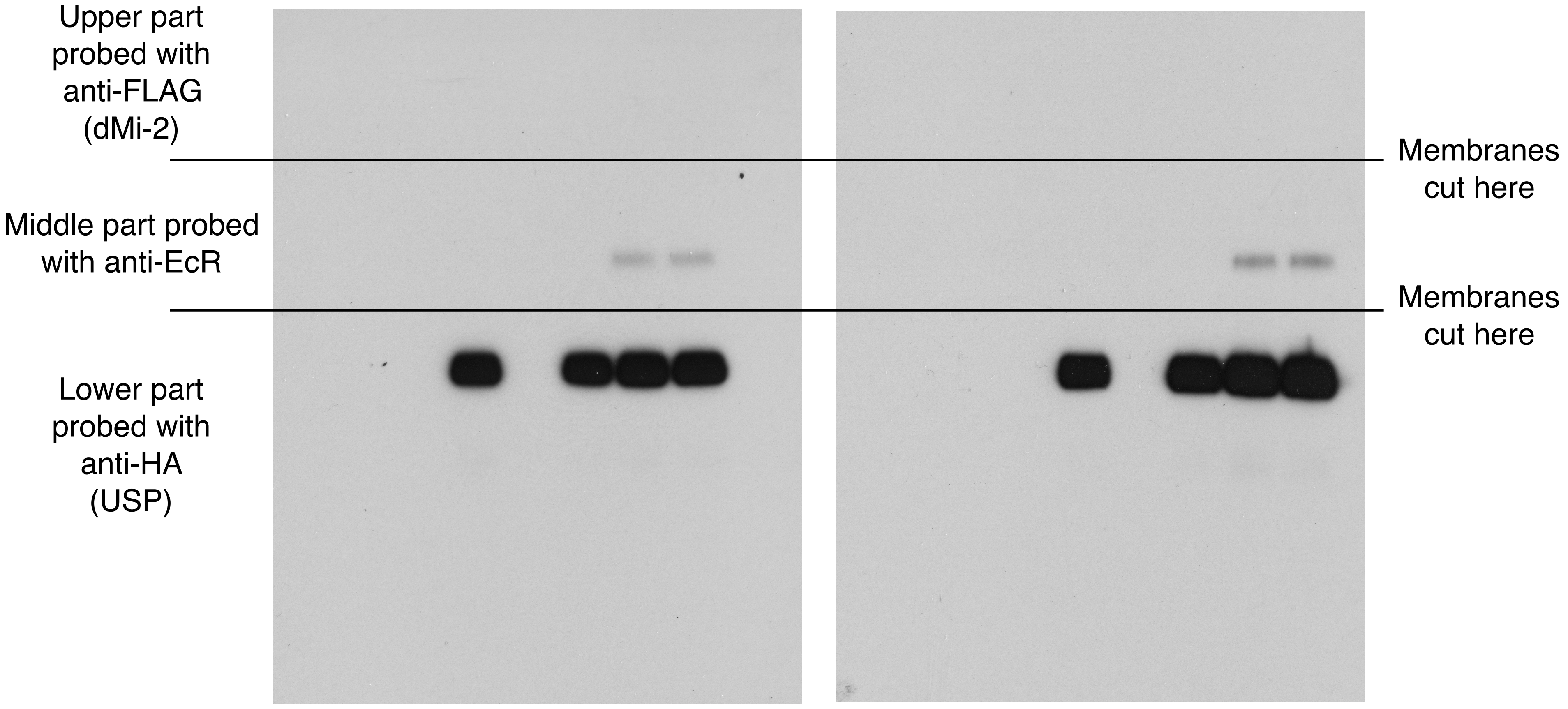

Figure 4C: Uncropped Western Blot, HA IP, bottom left

Figure 4C: Uncropped Western Blot, HA IP, bottom right

Supplementary Figure 4

Supplementary Table 1

RNAi T7 primer for dsRNA synthesis

**Oligo name**	**Sequence**
5’-T7-EcR	taatacgactcactatagggTACTCGCAGCGTTACGAAG
3’-T7-EcR	taatacgactcactatagggTACTCAACTGGACCGTGAG
5'-T7-EGFP	gaattaatacgactcactatagggaGAGCTGGACGGCGACGTAA
3'-T7-EGFP	gaattaatacgactcactatagggagACTTGTACAGCTCGTCCATG
5'-T7-Mi-2(2)	taatacgactcactatagggTTAACTCGCTGACCAAGGCT
3'-T7-Mi-2(2)	taatacgactcactatagggATATCGTTGTGGGGATTCCA
5'-T7-USP	taatacgactcactatagggAAAAATGTCGACGCGAAAAA
3'-T7-USP	taatacgactcactatagggTGGAGCAGGTTGCAGTTTC
5'-T7-ISWI	taatacgactcactatagggAGGCGCATCGTATCAAGAAC
3'-T7-ISWI	taatacgactcactatagggCGTTGCATCTTGGATAGACC

Supplementary Table 2

RT-qPCR primer

Oligo name	Sequence
Br-C_fwd	TACAACCGCACCATCCAGT
Br-C_rev	ATGCGTTACGATGCGATG
CR44742_fw	CAATGACACTTGGGCATGG
CR44742_rev	TGTGGACGTGGAATTGGAT
CR44743_fw	TTTTGTAAAAACCTTAAATGCCACT
CR44743_rev	TTGTGCTAACTAATCTGCGTACAGT
E23_fwd	GCTCATGCTCCTGGATGAA
E23_rev	CGCTGATCACGATGGTCTT
Hr4_fwd	TGCTCTCCCACATACCAGAGA
Hr4_rev	CACGAAGGGCACATAGAACA
Iswi_fwd	AAAGGATGTGGCCGATCA
Iswi_rev	AGGCATCGAAGCGAAAGAT
let-7_fwd	AGGTGCGATCTAGTGTGCCGTCTC
let-7_rev	TTAGGGCAAGCTCTGTTGTCCGAA
Mi-2_fwd	CGATTCTCTCCCGACTGG
Mi-2_rev	CAATGTTGTGCCCTGGAAT

Rp49_fwd	TGTCCTTCCAGCTTCAAGATGACCATC
Rp49_rev	CTTGGGCTTGCGCCATTTGTG
vrille_fwd	ATGAACAACGTCCGGCTATC
vrille_rev	CATATTTGCCCAGACTGTCG
EcR-all_fwd	CAGGAGGACCAGATCACGTT
EcR-all_rev	TTCGCGAAGAATATTGAGTCC
usp_fwd	GCTCCTTTGAGCGACGAT
usp_rev	CGAGAAGCTCTGGTTGAGGA

Supplementary Table 3

ChIP-qPCR primer

Oligo name	Sequence
Broad_ChIP1_fwd	GCCGGCAATATTAGAAGTTCG
Broad_ChIP1_rev	ATTGGATTGGATGGTGCAG
Broad_ChIP2_fwd	AACTTTAGAGGCAGCCCACA
Broad_ChIP2_rev	AGGTAGCAGGGGTACAGTGG
Broad_ChIP3_fwd	TGCCCACACAGACACACAG
Broad_ChIP3_rev	GCCAACTGTGCCTAACTGGT
Broad_ChIP4_fwd	TTCGCAGTCGCTGTTTTCT
Broad_ChIP4_rev	AACAACCTGACGGCGTAGAC
Broad_ChIP5_fwd	CACAGAAGGAAGAAGCAGCA
Broad_ChIP5_rev	CGGGACTGGCAAATTTCTT
Broad_ChIP6_fwd	GCCAGCTGGAGAAAGGTG
Broad_ChIP6_rev	GATTCCCATTCCCACTGATACT
Broad_ChIP7_fwd	GCGCGTCTCTGGACTCAC
Broad_ChIP7_rev	TGGCCAATACTCACGCTGT
Broad_ChIP8_fwd	CAATGATGAAAGCGCAAGC
Broad_ChIP8_rev	CACAGTTTTTCCATTTGCCTAA
Broad_ChIP9_fwd	GGGGCGTTTTGGTAGAACTAA
Broad_ChIP9_rev	TGGTTAGGCATAGACGTGTCC
intergenic2R_fwd	TGCTGACTGCCATCAAATTC
intergenic2R_rev	TACTTGCTGTGACGGCTTTG
vrille_ChIP1_fwd	GATTTAAAAGCCGCCAACTG
vrille_ChIP1_rev	GAGCTGTTATCACAACTGCAAAG

vrille_ChIP2_fwd	TGTGGACGTGGAATTGGAT
vrille_ChIP2_rev	CAATGACACTTGGGCATGG
vrille_ChIP3_fwd	GCCGCTTGTCGCTTATGTA
vrille_ChIP3_rev	TTCTGAGACTGCTTCCTTTGC
vrille_ChIP4_fwd	GGGTTTTATCGCTGTTGCAT
vrille_ChIP4_rev	CATACGCCCCATGGGTTA
vrille_ChIP5_fwd	TCTCTTTGGCTCCCACTCTG
vrille_ChIP5_rev	AAGCGGTAATAGCCAGCAAA
vrille_ChIP6_fwd	GTTTCTTCTGCCCCAATGC
vrille_ChIP6_rev	CCTCTTTGGCCGAAAAATCT
vrille_ChIP7_fwd	TGTGTGTGTGTGATTGTGCTG
vrille_ChIP7_rev	AGAGGGAGCGAGAATTAGACG
vrille_ChIP8_fwd	AGGCCAATGTGGTAACCAGT
vrille_ChIP8_rev	TGGCCACCTCGGACTCTA
vrille_ChIP9_fwd	TTGTAGGGTATCCTGTCCGAAT
vrille_ChIP9_rev	GAAGATTTAGCATTTTGATGGATTT

Supplementary Table 4

Primer for MNase protection assay

Oligo name	Sequence
**vrille2_MN1_fw**	**GGAATTGGATGTTGCTTCTGGT**
**vrille2_MN1_re**	**AAGTCTTTGGCTGGCGTCGC**
vrille2_MN2_fw	CGCGGGCCCGTTCTGCCCAT
vrille2_MN2_rev	TTACCGCACGTCCTTTATG
vrille2_MN3_fw	TAACCTTGAAAAGTTTAACTT
vrille2_MN3_rev	CACATGATCCGAGTACATCG
vrilleRE_MN1_fw	AAGCAATTGCGTCGACTGAGC
vrilleRE_MN1_re	GGGTTGTTGTTGGGGATGATGTTG
**vrilleRE_MN2_fw**	**GTGAAATTTCTGTGCGGCGGC**
**vrilleRE_MN2_re**	**TAACGACCAACGGCCGCGCCT**
vrilleRE_MN3_fw	GGCAAAAGATCGAGAATTTC
vrilleRE_MN3_re	TGTGAGCAATTGCATATTTC
**vrilleRC_MN1_fw**	**ACCGCTTATGTTAAGTGATT**
**vrilleRC_MN1_re**	**ACTTAGCCGTATTTATGACTC**
vrilleRC_MN2_fw	GGATTTCTCAGCCGTTCTGA

vrilleRC_MN2_re	ATTTGATTTTGGGGTCTATTG
vrilleRC_MN3_fw	TATGTGTCATAAGGTGAAAC
vrilleRC_MN3_re	CGACACTATGAAGCCCAGTT
vrille6_MN1_fw	GTACAAAATTTCGGTTTCGT
vrille6_MN1_re	GGTTTTGGAAGAACCCCCAA
**vrille6_MN2_fw**	**GAATTGCCCGGGTGGCGGGG**
**vrille6_MN2_re**	**GCTTACAACTTTCACACCGCA**
vrille6_MN3_fw	TGTGCTAGACGTTTCGATGTTG
vrille6_MN3_re	CAACAAGCATTGGGGCAGAAG
**vrilleRA_MN1_fw**	**AATGCTTTTACAAATTCAATTG**
**vrilleRA_MN1_re**	**AACCCGATCGGCTGTATATTA**
vrilleRA_MN2_fw	GACTATACTACCAAACCATATA
vrilleRA_MN2_re	CTGCGCTTGATGCACTTGGCC
vrilleRA_MN3_fw	TCGAGCCGTTAAAAGCATTT
vrilleRA_MN3_re	GAAAATTGTTTTTCAAACACTTG
**vrilleEx_MN1_fw**	**CTATCGCGTCGGGCCTGCTCACC**
**vrilleEx_MN1_re**	**TCGTGCTCCTGCTCATCATC**
vrilleEx_MN2_fw	CATCGGGAGCTGTTTCCGGCG
vrilleEx_MN2_re	GCTTGAGGGGCAGACAGTTGT
vrilleEx_MN3_fw	AGTCCGCAGCAGGGCAGCGAT
vrilleEx_MN3_re	TGAGAGTAGCGCCGTGGC
vrille3'_MN1_fw	CCAGCAAAGGTCTTTTACTGCC
vrille3'_MN1_re	TGTGTAATAGATTAACTTGC
vrille3'_MN2_fw	ATACACAATTATTGTATATCAGC
vrille3'_MN2_re	TAACTCATTCTGCAATATGCGAT
**vrille3'_MN3_fw**	**CACCATCATCGCACGAGCTTA**
**vrille3'_MN3_re**	**TAATCTCTTTACCACTCGACG**

For each theoretical nucleosome, three overlapping oligo pairs were designed. Oligo pairs depicted in bold were selected as they showed to be protected from MNase digest according to qPCR results.
